# Integrated nutrient profiling and metabolomic analysis reveal nutritional enhancement in autotetraploid brown rice

**DOI:** 10.3389/fpls.2026.1972414

**Published:** 2026-10-14

**Authors:** Yingkai Wang, Xiaoyu Wang, Changjiang Zhang, Zhao Zhang, Weilong Meng, Liangyu Chen, Minghong Xu, Zitian Leng, Yan Ye, Ningning Wang, Jian Ma

**Affiliations:** 1 Faculty of Agronomy, Sanjiang Laboratory, Jilin Agricultural University, Changchun, China; 2 Rice Research Institute, Liaoyuan Academy of Agricultural Sciences, Liaoyuan, China

**Keywords:** autotetraploid rice, brown rice, metabolomics, nutritional quality, polyploidization

## Abstract

To investigate the effects of polyploidization on the nutritional quality of brown rice, three pairs of homologous diploid and autotetraploid rice lines with a japonica genetic background (GFD, 397, and T13) were examined for grain morphology and nutrient composition, and their metabolite profiles were analyzed using UPLC-MS/MS-based metabolomics. Compared with their diploid counterparts, all three autotetraploid lines produced larger grains. Among the 77 nutritional traits measured, resistant starch, crude fat, and crude protein contents were significantly increased. Protein fraction analysis indicated enhanced accumulation of major storage proteins, and most amino acids, particularly essential amino acids, were present at higher levels in autotetraploid brown rice. Vitamin A content increased significantly, several B vitamins showed an overall increasing trend whose magnitude varied among genotypes, whereas vitamins D content decreased. Overall, chromosome doubling altered nutrient composition through both lines consistent and lines dependent responses. Metabolomic profiling identified 1,211 metabolites, and the differentially abundant metabolites were characterized primarily by the upregulation of amino acids and their derivatives and the downregulation of certain flavonoids. KEGG enrichment analysis further showed that the differentially abundant metabolites were mainly enriched in pathways related to amino acid biosynthesis, suggesting that metabolite accumulation in these pathways may be associated with the increased protein and amino acid contents in autotetraploids, although the underlying mechanisms require further investigation. Collectively, these findings demonstrate indicate that polyploidization is associated with improvement in several nutritional quality of brown rice and provide a basis for the further evaluation and utilization of autotetraploid brown rice.

## 1 Introduction

Rice is the staple food for more than half of the global population and a cornerstone of dietary patterns across Asia (Kasote et al., 2022). With rising living standards and the increasing prevalence of chronic diseases, greater attention has been paid to nutrition and healthy diets (Saleh et al., 2019). Rice contains a wide range of nutritional components, among which starch is the most abundant, followed by proteins, lipids, vitamins, and phenolic acids (Yoshida et al., 2010).

Starch is primarily composed of amylose and amylopectin, and their relative proportions markedly influence its physicochemical properties and digestibility (Zhu et al., 2023; Liu et al., 2021). Amylose has a predominantly linear structure, is digested more slowly, and readily undergoes retrogradation to form resistant starch, thereby conferring a firmer texture and contributing to glycemic control (Liu et al., 2021). Based on its digestibility, starch can be classified into resistant starch (RS) and non-resistant starch (NRS) fractions: the former undergoes fermentation in the gut and has been associated with modulation of the intestinal microbiota, improved insulin sensitivity, and the prevention of metabolic disorders, whereas the latter is mainly hydrolyzed in the small intestine and serves as a major energy source (Zhu et al., 2023; Shen et al., 2022). In plants, glucose produced through photosynthesis is repolymerized via dehydration (condensation) reactions catalyzed by starch synthases, branching enzymes, and related enzymes, ultimately forming amylose and amylopectin (Tappiban et al., 2021). This process represents the biosynthesis of polysaccharides from monosaccharides and constitutes a major mechanism of energy storage in plants (Gurung et al., 2025).

Rice protein is a major storage protein in rice seeds and mainly consists of glutelins and prolamins. Its allergenicity is substantially lower than that of common food proteins derived from wheat and legumes, making it less likely to trigger allergic reactions (Shao, 2020; Shewry, 2007). In addition, rice protein exhibits high digestibility and a favorable amino acid profile, particularly rich in lysine and threonine, which are critical for infant growth and development, thereby contributing to its favorable amino acid balance and overall protein nutritional quality (Shao, 2020). Owing to these attributes, rice protein is considered a plant-derived protein source with high safety, good nutritional value, and desirable palatability. It is regarded as an ideal protein ingredient for the development of infant formula and functional foods (Shewry, 2007).

Rice lipids are rich in unsaturated fatty acids, with relatively high levels of polyunsaturated and monounsaturated fatty acids such as linoleic acid and oleic acid, which are important for maintaining the structural integrity of cell membranes, regulating blood lipid profiles, and supporting cardiovascular health (Zhou et al., 2024). In addition to proteins and lipids, rice provides a variety of essential micronutrients, including B vitamins, vitamin E, and small amounts of vitamin A (Liu et al., 2013). In addition, rice is rich in diverse bioactive constituents, including proanthocyanidins and other phenolic acid compounds (Goufo and Trindade, 2017). Therefore, further enhancing the nutritional value of rice and increasing the levels of functional components through cultivar improvement, optimized agronomic management, and improved processing strategies have substantial potential to improve global dietary quality and promote public health (Weh et al., 2024).

Polyploidization is a major driving force in biological evolution and speciation, as increases in genome copy number can substantially enhance genetic variation and environmental adaptability (Van Drunen and Johnson, 2022). Polyploidization also frequently reshapes plant morphological, physiological, and metabolic traits, such as increased organ size, enhanced photosynthetic capacity, and improved stress tolerance, thereby offering considerable value for crop improvement. Previous studies have shown that relative to corresponding diploid lines, tetraploid rice tends to have higher protein and amino acid contents but lower amylose content (Zhang and Song, 1992). Moreover, increased abundances of lipids, amino acid derivatives, and phenolic acids have also been reported in tetraploid rice (Wang et al., 2022).

More importantly, the influence of polyploidization on rice quality may extend beyond changes in morphology and yield-related traits to extensive reprogramming of metabolic pathways (Comai, 2005; Sattler et al., 2016). Increased genome dosage can alter gene expression patterns, enzyme activities, and metabolic fluxes, thereby affecting the biosynthesis and accumulation of both primary and secondary metabolites (Comai, 2005; Dhawan and Lavania, 1996). Such metabolite-level alterations are of particular importance because they are closely associated with nutritional value, eating quality, stress adaptation, and the accumulation of health-promoting compounds (Sattler et al., 2016; Dhawan and Lavania, 1996). However, compared with studies on agronomic performance and major nutritional constituents, the effects of polyploidization on the rice grain metabolome remain insufficiently characterized. Therefore, characterizing the metabolic consequences of polyploidization is essential for understanding how genome duplication translates into variation in grain quality and for facilitating the development of tetraploid rice with enhanced nutritional and functional value.

To systematically compare the nutrient composition and metabolite profiles of brown rice between diploid rice and their corresponding homologous autotetraploid lines, three diploid lines and their respective homologous autotetraploid counterparts were selected for analysis. Major nutritional components in brown rice were determined using chemical analytical methods, with crude protein including the Kjeldahl method for nitrogen determination. In parallel, untargeted metabolomic profiling was performed using ultra-performance liquid chromatography-tandem mass spectrometry (UPLC-MS/MS). By comparing diploid and homologous autotetraploid lines within each of three varieties backgrounds, this study aimed to evaluate ploidy-associated difference in nutrient composition and metabolite accumulation in brown rice, and to provide a basis for further evaluation and utilization of autotetraploid brown rice.

## 2 Materials and methods

### 2.1 Plant materials

Three japonica rice varieties, each represented by a diploid line and its corresponding autotetraploid line, were used in this study: GFD (GFD-2X/GFD-4X), Kirara 397 (397-2X/397-4X), and Tongfeng No. 13 (T13-2X/T13-4X). GFD-4X and T13-4X originated from spontaneous tetraploid plants identified in Teaching and Research Base of Jilin Agricultural University with their respective diploid populations on the basis of phenotype variation, e.g., markedly enlarged grains, thicker culms, and lager stomata. 397-4X was generated by 0.04% colchicine treatment three days of germinating seeds of Kirara 397 in Teaching and Research Base of Jilin Agricultural University. For all three putative tetraploids, ploidy was initially confirmed by karyotype analysis, in which chromosome numbers were counted in root-tip cells at metaphase, plants with 2n=4x=48 choromosomes were retained as tetraploids, whereas those with 2n=2x=24 or with aneuploid chromosome numbers were discarded (**Supplementary Figure 1**) (Leng et al., 2023). The confirmed tetraploid plants were subsequently advanced by single-plant selection and self-pollination for seven consecutive generations in the same field environment at the Teaching and Research Base of Jilin Agricultural University (Changchun, Jilin province, China). At each generation, plants showing off-type agronomic traits were discarded, and only phenotypically uniform plants with typical tetraploid characteristics were retained. By the seventh generation, the three autotetraploid lines were stable in ploidy and major agronomic traits.

### 2.2 Field experiment and sampling

The field experiment was conducted in the year of 2023 at the Teaching and Research Base of Jilin Agricultural University. Seeds that had been stored for five months after the previous harvest were sown in April, and seedlings were transplanted in May at a spacing of (30 cm × 20 cm), with one seedling per hill. The six lines were arranged in a randomized complete block design with three replicate plots per line, each plot consisted of 6 rows × 40 plants (14.4 m²). Field management, including fertilization, irrigation, and pest control, followed local standard practices and was identical for all plots. At maturity, seven plants were randomly selected from the center of each plot, avoiding border rows, and their grains were pooled to form one biological replicate. Thus, each line had three biological replicates, each derived from an independent plot. Grains were collected from the middle and upper portions of the panicles, excluding the apical spikelets, and only well-filled, fully mature seeds were used. For nutrient analysis, grains were dehulled and ground into powder, and 60 g of powder was used for each technical replicate, with three technical replicate per biological replicates. For metabolomic profiling, 1g of brown rice from each of the three biological replicates was used.

### 2.3 Determination of starch-related components

Starch-related components in diploid and autotetraploid rice were quantified using a microplate reader (EPOCH 2, BioTek Instruments). Key reagents used in the process included ethanol, ether, glacial acetic acid, and sodium acetate. Specifically, the contents of amylose, amylopectin, RS, and NRS were determined using commercial assay kits (ADS-W-DF009, M1103A, Suzhou Michy Biomedical Technology Co., Ltd., Suzhou, China).

### 2.4 Determination of crude fat content

The crude fat content of diploid and autotetraploid rice was determined using a fat analyzer (Hanon SOX406, China) with low-boiling-point petroleum ether as the solvent. A modified Soxhlet extraction method (residue method) was employed for the analysis. Crude fat was removed from the samples via reflux extraction with petroleum ether, and the fat content was calculated as the weight difference between the initial sample and the dried residue (Khalid et al., 2021).

### 2.5 Determination of protein and amino acid content

The crude protein content of diploid and autotetraploid brown rice was quantified using an automatic Kjeldahl analyzer (Hanon K9860, China). The analysis utilized sulfuric acid, 30% hydrogen peroxide, 40% sodium hydroxide, 2% boric acid, a methyl red-bromocresol green mixed indicator, and a 0.1008 mol/L standard acid solution. The total nitrogen content was determined via the Kjeldahl method, and crude protein was calculated using a conversion factor of 6.25 (Khalid et al., 2021). Additionally, the four protein fractions: albumin, globulin, prolamin, and glutenin, were sequentially extracted using distilled water, 10% NaCl, 70% ethanol, and 0.2% NaOH, respectively. The protein content of each fraction was subsequently determined using the Kjeldahl apparatus (Khalid et al., 2021).

The profiles of 17 hydrolyzed amino acids and 6 free amino acids were analyzed using an Agilent 1100 High-Performance Liquid Chromatography (HPLC) system equipped with an Amethyst C18-H column (250 mm × 4.6 mm, 5μm). Sample preparation and derivatization employed HPLC-grade acetonitrile, hydrochloric acid, norleucine (as an internal standard), triethylamine, phenyl isothiocyanate (PITC), n-hexane, glacial acetic acid, and anhydrous sodium acetate.

### 2.6 Determination of vitamin content

The vitamin content of diploid and autotetraploid rice was analyzed using HPLC systems (Waters 2695 and Agilent 1100). Separation was achieved using an Ultimate XB-C30 column (250 mm × 4.6 mm, 5μm) and a Compass C18(2) reversed-phase column (250 mm × 4.6 mm, 5μm). Sample preparation involved the use of an ultrasonic cleaner and a high-speed refrigerated centrifuge. A comprehensive panel of vitamins was quantified, including VA, the B-complex (B1, B2, B3, B5, B6, B7, B9, B12, B17), VD2, VD3, VK, and tocopherols (α-, β-, γ-, and δ-tocopherol). The analysis utilized high-purity standards and reagents, including HPLC-grade methanol, acetonitrile, petroleum ether, ethanol, potassium hydroxide, phosphoric acid, hydrochloric acid, and butylated hydroxytoluene (BHT).

### 2.7 Determination of phytochemicals and metabolites

Total Sterols, phytic acid, and other components: the contents of total sterol, phytic acid, proanthocyanidin, β-glucan, carotenoids, and alkaloids were quantified using a microplate reader. Commercial assay kits (M0148A, M0125A, M0121A, ADS-W-TDX045, M1901A, M0122A, Suzhou Michy Biomedical Technology Co., Ltd., Suzhou, China) were used for these analyses, with concentrated sulfuric acid and other requisite reagents used according to the manufacturer’s instructions.

Phytosterols: ergosterol, stigmasterol, campesterol, and β-sitosterol were analyzed using a Waters 2695 High-Performance Liquid Chromatography (HPLC) system. Separation was achieved using an Ultimate XB-C30 column (250 mm × 4.6 mm, 5μm) and a Compass C18(2) reversed-phase column (250 mm × 4.6 mm, 5μm). Standard solutions of each sterol and HPLC-grade methanol were utilized for quantification.

Inositol: Inositol content was determined via Gas Chromatography-Mass Spectrometry (GC-MS). Sample preparation involved a high-speed refrigerated centrifuge and extraction using chromatographic-grade methanol and petroleum ether. Trimethylchlorosilane (TMCS) was used as a derivatizing agent, and quantification was performed using an inositol standard.

γ-Aminobutyric Acid (GABA): GABA content was quantified using an Agilent 1100 HPLC system equipped with a Compass C18(2) reversed-phase column (250 mm × 4.6 mm, 5μm). Derivatization was performed using 4-dimethylaminoazobenzene-4-sulfonyl chloride (DABS-Cl) in the presence of anhydrous sodium acetate and sodium bicarbonate. HPLC-grade acetonitrile served as the mobile phase component.

Phenolic Acids and Flavonoids: a profile of eight phenolic compounds (ferulic acid, vanillic acid, naringenin, hesperetin, cinnamic acid, protocatechuic acid, p-coumaric acid, and apigenin) was determined using HPLC systems (Agilent 1100 and Waters 2695). Separation was performed on a Compass C18(2) column. The analysis employed specific standards for each compound, HPLC-grade methanol, anhydrous sodium acetate, and phosphoric acid.

Catechins: Catechin profiles were analyzed using an Agilent 1100 HPLC system with a Compass C18(2) column. The analytes included gallocatechin (GC), epigallocatechin (EGC), catechin (C), epicatechin (EC), epigallocatechin gallate (EGCG), gallocatechin gallate (GCG), epicatechin gallate (ECG), and catechin gallate (CG). Quantification relied on respective standards and HPLC-grade solvents, including methanol and acetonitrile.

### 2.8 Metabolome profiling

Metabolomic profiling was conducted using UPLC-MS/MS (ExionLC™ AD, SCIEX). Freeze-dried samples were ground (30 Hz, 1.5 min) and extracted (50 mg) with 1,200 μL of 70% (v/v) methanol in water containing internal standards, at -20 °C. The extracts were vortexed intermittently (30 s every 30 min, 6 times), centrifuged (12,000 rpm, 3 min), and filtered (0.22 μm). Chromatographic separation utilized 0.1% aqueous formic acid and acetonitrile at a flow rate of 0.35 mL/min (40 °C, 2 μL injection). MS/MS detection operated with source temperatures at 550 °C and ion spray voltages of +5500/−4500 V. Metabolite identification relied on the Metware Database (MWDB), while quantification was performed in MRM mode using Analyst 1.6.3 software.

### 2.9 Statistical analysis

Nutritional component data were analyzed by first averaging the three technical replicates within each biological replicate. Three biological replicates derived from independent plots were used as the statistical units for each line (n=3). Data are presented as the mean ± standard deviation (SD). The primary comparisons in this study focused on differences between each autotetraploid line and its corresponding isogenic diploid line within each of the three varieties. Two-tailed independent-samples *t*-tests were performed for pairwise comparisons within each genotype. In addition, one-way analysis of variance (ANOVA), followed by Tukey’s honestly significant difference (HSD) test, was used to compare differences among the six lines (*p* < 0.05). The consistency of ploidy-associated changes across the three varieties was evaluated by examining the direction and statistical significance of tetraploid-diploid differences within each variety. No formal inference regarding ploidy effects was extended beyond the tested lines. All statistical analyses were performed in R (version 4.5.2) using the stats, agricolae (version 1.3-7), and multcomp (version 1.4-30) package Sankey diagrams, heatmaps, correlation plots and other visualizations were generated in R using plotly (version 4.12.0), HeatR (version 1.1.0), pheatmap (version 1.0.13), and corrplot (version 0.95) packages.

Metabolomic data (six lines × three biological replicates, 18 samples in total) were log_2_-transformed and unit-variance scaled prior to analysis. Principal component analysis (PCA) was first performed to assess the consistency of biological replicates and the overall distribution of samples by genotype and ploidy (**Supplementary Figure 3D**). Orthogonal partial least squares-discriminant analysis (OPLS-DA) was then conducted for each diploid line versus its corresponding autotetraploid line, as well as for all diploid versus all autotetraploid samples. Model quality was evaluated by R²X, R²Y, and Q² values, and validity was confirmed by a 200-iteration permutation test (Q² intercept < 0.05, *p* < 0.05) (**Supplementary Figure 5**). Differentially accumulated metabolites (DAMs) were defined as those with VIP > 1, |log_2_FC| ≥ 1. All analyses were performed on the Metware Cloud platform.

KEGG pathway enrichment analysis of DAMs was conducted using the Metware Cloud platform (https://cloud.metware.cn/) based on a hypergeometric test. Metabolomic data were visualized using an online bioinformatics platform (http://bioinformatics.com.cn/), and trends in DAM abundance were plotted using Origin 2022 (OriginLab, USA).

## 3 Results

### 3.1 Difference analysis of nutritional contents in diploid and autotetraploid brown rice

Clear differences in grain morphology were observed between each autotetraploids line and its corresponding diploid progenitor (GFD-4X vs. GFD-2X, 397-4X vs. 397-2X, and T13-4X vs. T13-2X). Brown rice of all three autotetraploid lines had larger grains than that of their diploid progenitors (**Figure 1A**). Consistently, the thousand-grain weight of the three autotetraploid lines was significantly higher than that of their respective diploids, by 51.60%, 40.61%, and 44.91% for GFD, 397, and T13, respectively (all *p* < 0.05) (**Supplementary Table 1**).

**Figure 1:**
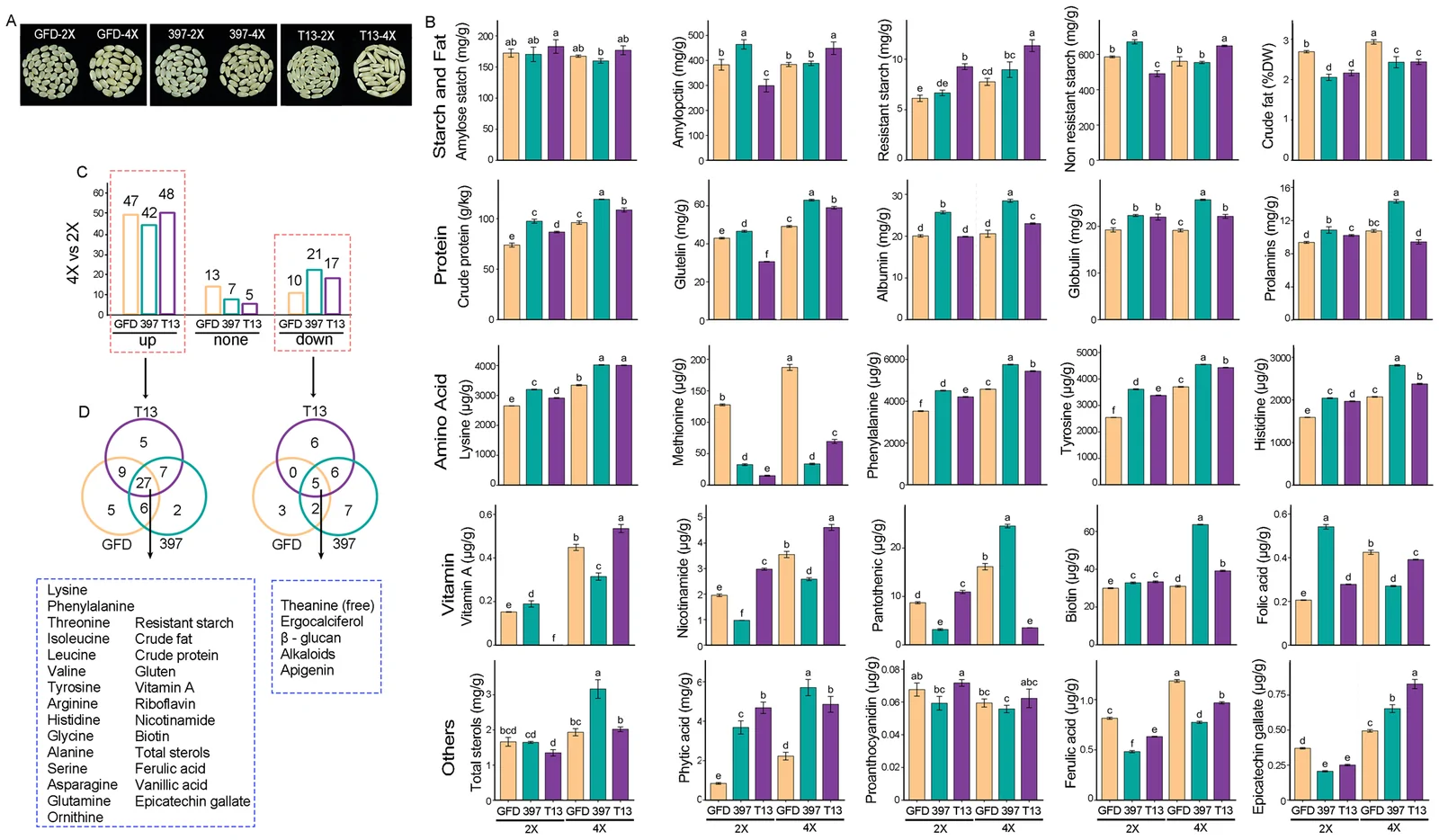
Impact of polyploidization on the morphology and nutritional profile of brown rice. **(A)** Phenotypic comparison of grain morphology between diploid and autotetraploid lines. **(B)** Quantification of key nutrients (starch, fat, protein, amino acids, vitamins, and other substances). Different letters denote significant differences (*p* < 0.05). **(C)** Statistics of upregulated, downregulated, and non-significant nutrient changes in autotetraploids relative to diploids. **(D)** Venn diagram depicting the overlap and specificity of modulated nutrients across the three genotypes.

A total of 77 nutritional components, covering starches, fats, proteins, amino acids, vitamins, and other compounds, were quantified in the six lines (**Figure 1B**; **Supplementary Figure 2**; **Supplementary Table 2**). Seven of them were excluded from the statistical comparison because they were below the limit of quantification in one or more lines, leaving 70 components for analysis. All 70 differed significantly between the autotetraploid line and its diploid progenitor in at least one variety. Of these, 32 changed significantly and in the same direction in all three backgrounds, whereas the remaining 38 varied in significance or direction among backgrounds (**Figure 1**), indicating that chromosome doubling altered a core set of nutritional traits consistently but that a part of the response depended on line-specific genetic background.

To minimize measurement error and enable direct comparison across samples, all nutrientional components were quantified and expressed as concentrations on a dry weight (DW) basis (mg/g or µg/g), except for crude fat, which was determined as a percentage (w/w). Among the six lines (GFD-2X, 397-2X, T13-2X, GFD-4X, 397-4X, and T13-4X), amylose concentrations were approximately 172.39, 170.36, 182.97, 167.40, 159.79, and 176.91 mg/g, respectively (**Supplementary Table 2**). Compared with their corresponding diploid progenitors, amylose concentrations decreased by 2.9% in GFD-4X, 6.2% in 397-4X, and 3.3% in T13-4X (**Figure 1B**; **Supplementary Table 2**). By contrast, shifts in amylopectin were lines dependent: amylopectin increased significantly by 50.08% in T13-4X (*p* < 0.05) and decreased by 16.36% in 397-4X (*p* < 0.05), whereas the difference in GFD-4X relative to GFD-2X (+0.34%) was not statistically significant (*p* < 0.05). RS concentrations increased significantly in all three autotetraploids, by 26.60% in GFD-4X, 34.44% in 397-4X, and 22.42% in T13-4X (all *p* < 0.05). NRS decreased significantly by 4.36% in GFD-4X and 17.44% in 397-4X, but increased by 12.93% in T13-4X (**Figure 1B**; **Supplementary Table 2**). Crude fat content increased significantly by 8.98%, 18.24%, and 12.75% in GFD-4X, 397-4X, and T13-4X, respectively (all *p* < 0.05) (**Figure 1B**; **Supplementary Table 2**).

Protein profiling indicated that autopolyploidization significantly increased crude protein content across all three autotetraploids lines, with increases exceeding 20% (+22.25% to +30.17%, *p* < 0.05) (**Figure 1B**; **Supplementary Table 2**). Quantification of Osborne protein fractions further revealed marked increases in glutelin and albumin in the autotetraploids (+2.61% to +92.59%), with the most pronounced glutelin increase observed in T13-4X (+92.59%, *p* < 0.05). Globulin increased significantly only in 397-4X (+15.06%, *p* < 0.05), remaining statistically unchanged in the other two pairs. In contrast, prolamin exhibited genotype-dependent responses, increasing significantly in GFD-4X (+14.78%, *p* < 0.05) and 397-4X (+31.83%, *p* < 0.05), but declining significantly in T13-4X (-7.67%, *p* < 0.05) (**Figure 1B**; **Supplementary Table 2**).

Amino acid profiling showed that all essential and semi-essential amino acids were higher in the autotetraploids than in their corresponding diploids, with the total essential amino acids increasing by more than 20% (*p* < 0.05), the only exception was the free non-protein amino acid theanine, which was consistently reduced (*p* < 0.05) (**Supplementary Figure 2**; **Supplementary Table 2**). We used p < 0.05 as the criterion for determining statistically significant differences in nutrient levels, while also calculating FDR-corrected p values to account for multiple comparisons (**Supplementary Table 2**). Notably, although some nutrients failed to pass FDR correction, their consistent directional changes across groups retained informative value for understanding the relevant nutrient metabolism pathways, though these findings warrant further validation.

Non-essential amino acids also exhibited an overall increase (**Figure 1B**; **Supplementary Figure 2**; **Supplementary Table 2**). These results indicate that chromosome doubling improves the amino acid composition of brown rice in the lines examined. The magnitude and significance of these increases were nevertheless lines dependent: lysine, methionine, phenylalanine, tyrosine, and histidine differed significantly between ploidy levels in the GFD and 397 backgrounds, whereas methionine did not differ significantly in T13 (**Figure 1B**; **Supplementary Figure 2**; **Supplementary Table 2**).

Vitamin profiling revealed marked increases in vitamin A and vitamins E in the autotetraploids: vitamin A increased by 53.49%-194.56%, and vitamin E increased by 79.49% on average (up to 109.99%) (all *p* < 0.05). In contrast, vitamins D levels decreased by more than 14% in all three pairs (*p* < 0.05). For B vitamins, VB3, VB7, and VB2 were consistently and significantly higher in autotetraploids, by 55.00%-166.36%, 3.40%-93.87%, and more than 33.24%, respectively (all *p* < 0.05). VB5 was strongly lines dependent, increasing in GFD-4X and 397-4X (+85.42% and +672.90%, respectively) but decreasing significantly in T13-4X (-67.57%) (all *p* < 0.05). VB6 and VB9 also differed significantly between ploidy levels, by -88.32% to +6.24% and -50.04% to +106.18%, respectively (**Figure 1B**; **Supplementary Figure 2**; **Supplementary Table 2**).

Among the other nutritional components quantified, phytic acid, proanthocyanidins, ferulic acid, and epicatechin gallate were significantly elevated in all three autotetraploids, with phytic acid increasing by 3.74%-163.88% (**Figure 1B**; **Supplementary Table 2**). Total sterols increased by at least 16% (*p* < 0.05), but changes in individual sterol species were lines dependent: ergosterol increased in GFD-4X and decreased in 397-4X and T13-4X, whereas stigmasterol showed the opposite pattern. Comparable genotype-specific responses were observed for naringenin, protocatechuic acid, catechin, and epigallocatechin gallate (opposite trends in the GFD group versus the other two backgrounds), for β-sitosterol, cinnamic acid, and p-coumaric acid (distinct trends in the 397 group versus GFD and T13), and for inositol, GABA, and carotenoids (distinct trends in the T13 group versus the other two backgrounds) (**Supplementary Figure 2**; **Supplementary Table 2**).

To further dissect nutritional differences between diploids and autotetraploids brown rice, we performed a Venn analysis of 70 nutrients (**Figure 1C**). Relative to their corresponding diploid progenitors, 47, 42, and 48 components increased significantly increased in GFD-4X, 397-4X, and T13-4X, respectively, whereas 10, 21, and 17 nutrients were significantly decreased, and 13, 7, and 5 showed no significant change (**Figure 1C**). Of the 32 components that changed concordantly in all three pairs, 27 were consistently increased, including resistant starch, crude protein, crude fat, 15 amino acids, and vitamin A, whereas 5 were consistently decreased, namely theanine, ergocalciferol, β-glucan, alkaloids, and luteolin (**Figure 1D**, the full list is provided in **Supplementary Table 2**).

### 3.2 Widely targeted metabolic profiling of diploid and autotetraploid brown rice based on UPLC-MS/MS

Widely targeted metabolomics was applied to systematically characterize metabolic differences between diploid brown rice and the corresponding autotetraploid lines. Metabolites were identified and quantified on an UPLC-MS/MS platform coupled with an in-house database, with multiple reaction monitoring (MRM) used for quantification. Total ion chromatograms (TICs) showed substantial overlap among samples, and quality control (QC) samples exhibited highly consistent peak intensities and retention times across repeated injections, indicating excellent instrumental stability (**Supplementary Figures 3A, B**).

To assess data dispersion, the coefficient of variation (CV) was calculated for each metabolite. More than 75% of the detected metabolites exhibited CV values < 0.3, indicating good data stability (**Supplementary Figure 3C**). Principal component analysis (PCA) showed that the cumulative explained variance exceeded 69.56%, with no outliers detected, QC samples clustered near the origin of the score plot, confirming good reproducibility (**Figure 2A**; **Supplementary Figure 3D**). Correlation analysis further demonstrated high consistency among biological replicates within each line (Pearson correlation, r ≥ 0.9, Q² ≥ 0.9, *p* < 0.05) (**Supplementary Figure 4**), and hierarchical cluster analysis (HCA) corroborated these results (**Figure 2C**).

**Figure 2:**
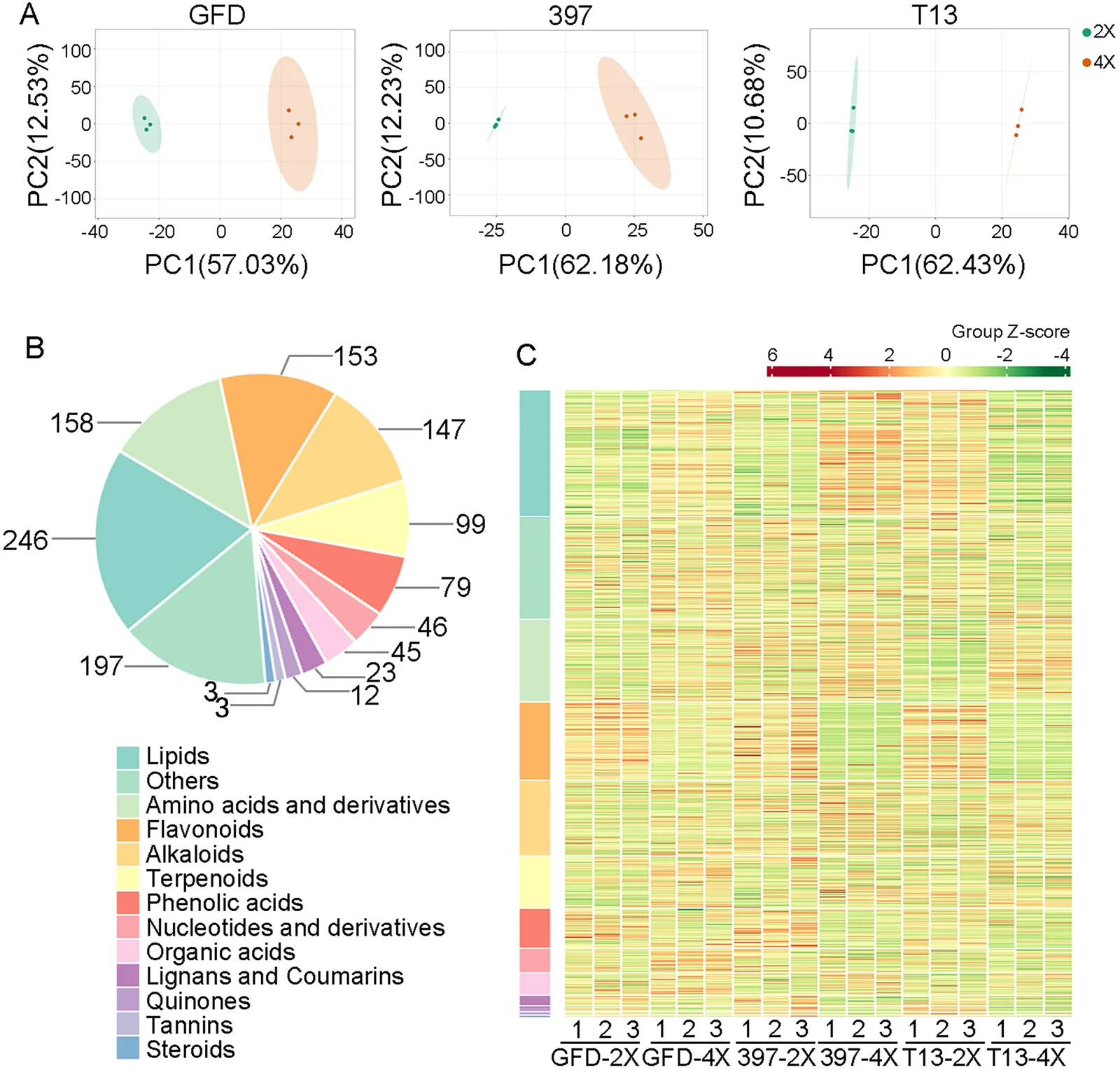
Overview of metabolic variations between diploid and autotetraploid brown rice. **(A)** Principal component analysis (PCA) showing the separation between diploid and autotetraploid lines. **(B)** Pie chart displaying the classification of detected metabolites. **(C)** Hierarchical cluster analysis (HCA) heatmap illustrating the overall metabolic clustering of the three rice genotypes.

To reduce the influence of variables weakly related to group discrimination, OPLS-DA was applied to remove orthogonal variation before screening for differentially accumulated metabolites (DAMs). Models were built on the Metware Cloud platform. The three pairwise comparisons (GFD-4X vs. GFD-2X, 397-4X vs. 397-2X, and T13-4X vs. T13-2X) all yielded models with Q² > 0.95, permutation tests produced intercepts < 0, indicating robust performance without overfitting (**Supplementary Figure 5**).

A total of 1,211 metabolites were detected in the diploid and autotetraploid rice lines and classified into 13 primary and 50 secondary categories. They comprised 246 lipids (20.3%), 158 amino acids and derivatives (13.0%), 153 flavonoids (12.6%), 147 alkaloids (12.1%), 99 terpenoids (8.2%), 79 phenolic acids (6.5%), 46 nucleotides and derivatives (3.8%), 45 organic acids (3.7%), 23 lignans and coumarins (1.9%), 12 quinones (1.0%), 3 tannins (0.2%), 3 steroids (0.2%), and 197 other compounds (16.3%) (**Figure 2B**; **Supplementary Tables 3**, **4**).

Across the six sample groups (GFD-2X, GFD-4X, 397-2X, 397-4X, T13-2X, and T13-4X), metabolites of all major classes were consistently detected in every biological replicate with comparable class distributions. The metabolite profiles were highly consistent among replicates, providing a reliable basis for the subsequent between-group comparisons (**Figure 2C**).

### 3.3 Analysis of the differential metabolites between diploid and autotetraploid brown rice

Using the OPLS-DA model, DAMs were screened with thresholds of VIP > 1 and fold change (FC) > 2 or FC < 0.5, yielding 270, 349, and 318 DAMs in the GFD, 397, and T13 diploid-autotetraploid pairs, respectively (**Figure 3A**, **Supplementary Table 4**). Compared with GFD-2X, 143 metabolites showed higher relative abundance in GFD-4X, whereas 127 showed lower abundance (**Figure 3A**). In the 397 pair, 137 metabolites were increased and 212 were decreased in 397-4X (**Figure 3A**). In the T13 pair, 134 metabolites were more abundant and 184 were less abundant in T13-4X (**Figure 3A**). The remaining 941, 862, and 893 metabolites in the GFD, 397, and T13 comparisons, respectively, did not meet the criteria for differential accumulation (**Figure 3A**).

**Figure 3:**
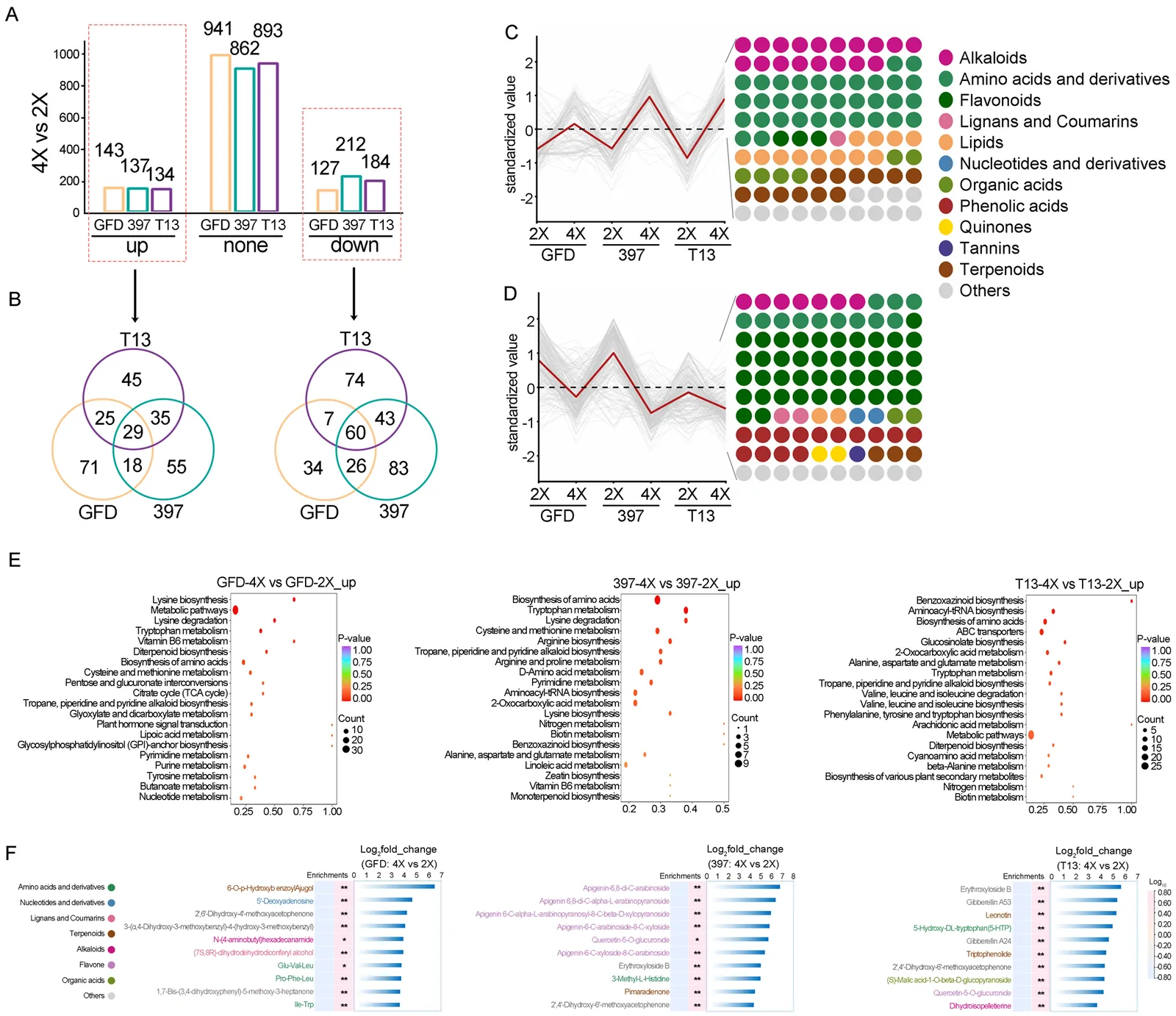
Analysis of differential metabolites (DAMs) in autotetraploid brown rice. **(A)** Number of up- and downregulated DAMs in each group. **(B)** Venn diagram showing common and specific DAMs among the three genotypes. **(C)** K-means clustering of upregulated metabolite profiles. **(D)** K-means clustering of downregulated metabolite profiles. **(E)** KEGG enrichment analysis of the identified up-regulated DAMs. **(F)** Heatmap of the top 10 upregulated metabolites with the highest Log_2_FoldChange. Asterisks denote significance (*p < 0.05, **p < 0.01).

Venn diagram analysis showed that 29 metabolites were commonly up-regulated in the autotetraploids relative to their diploid progenitors in all three genetic backgrounds, most of which were amino acids and their derivatives, whereas 60 metabolites were commonly down-regulated in autotetraploid brown rice (**Figure 3B**; **Supplementary Table 5**).

Many DAMs also showed coordinated variation. The relative abundances of DAMs identified in each comparison were subjected to unit variance (UV) scaling and then to K-means clustering. Several clusters displayed consistent trends across the three pairs (**Figures 3C, D**; **Supplementary Figure 6**). Two representative clusters, showing overall up- or down-regulation in the autotetraploids, were selected for further analysis, together they encompassed 12 metabolite categories. The DAMs with higher relative abundances in autotetraploid brown rice were mainly amino acids and their derivatives (**Figure 3C**), whereas those with lower relative abundance were predominantly flavonoids (**Figure 3D**).

To clarify the metabolic processes affected by chromosome doubling, KEGG enrichment analysis of the DAMs was performed, and the top 20 pathways were shown (**Figure 3E**; **Supplementary Table 6**). In all three comparison groups, up-regulated metabolites were mainly enriched in pathways related to amino acid biosynthesis and metabolism, such as “Biosynthesis of amino acids”, and in vitamin-related pathways, including “Vitamin B6 metabolism”. Lipid-related enrichment was genotype specific, involving “Butanoate metabolism”, “Linoleic acid metabolism”, and “Arachidonic acid metabolism” in the GFD, 397 and T13 pairs, respectively. Comparable genotype-specific patterns were observed for carbohydrate- and other amino acid-related pathways: In the GFD pair, up-regulated metabolites were specifically enriched in “Pentose and glucuronate interconversions” and “Citrate cycle (TCA cycle)”. In the 397 pair, in “Arginine biosynthesis” and “Zeatin biosynthesis”, and in T13 pair, in “Valine, leucine and isoleucine biosynthesis” and “Biosynthesis of various plant secondary metabolites”. These results indicate that, despite background-specific difference, up-regulated DAMs were consistently enriched in amino acid biosynthesis and metabolism in all three pairs.

The enriched pathways of down-regulated metabolites were analyzed in the same way (**Supplementary Figure 7**). Down-regulated DAMs shared by all three pairs were mainly enriched in “Biosynthesis of secondary metabolites and flavone” and “Flavonoid biosynthesis”. Genotype-specific enrichment was also observed: in the GFD pair, in “Tropane, piperidine and pyridine alkaloid biosynthesis”, in the 397 pair, in “Arachidonic acid metabolism”, and in the T13 pair, in “Betalain biosynthesis”.

To link pathway enrichment with the most strongly responding metabolites, the 10 most up-regulated DAMs (ranked by fold change) in each pair were compared in a heat map. These metabolites differed markedly among the three pairs and fell into eight categories: amino acids and derivatives, nucleotides and derivatives, lignans and coumarins, terpenoids, alkaloids, flavonoids, organic acids, and others (**Figure 3F**). Amino acid metabolites and their derivatives were elevated in all three pairs, with the most pronounced increase in the GFD pair.

Collectively, these findings indicate that chromosome doubling reshapes the metabolite profile of brown rice, mainly through higher accumulation of amino acids and their derivatives and lower accumulation of flavonoids, whereas changes in other metabolite classes depended largely on the genetic background of the parental diploid line. As only three variety pairs were examined, these conclusions apply to the lines tested.

### 3.4 Differential accumulation and pathway enrichment of saccharides, lipids, and amino acids between diploid and autotetraploid brown rice

Metabolomic profiling detected 64 saccharide metabolites, 29 of which were mapped to 14 KEGG pathways (**Figure 4A**). Sankey plots and heat maps of relative metabolite abundance showed that these saccharide metabolites were mainly associated with major pathways, including “Glycolysis/Gluconeogenesis”, “Starch and sucrose metabolism”, and “Amino sugar and nucleotide sugar metabolism”. Further analysis revealed that although some saccharide metabolites in these pathways did not differ significantly between diploid and autotetraploid brown rice and showed no consistent trend, most showed higher relative abundance in autotetraploid brown rice.

**Figure 4:**
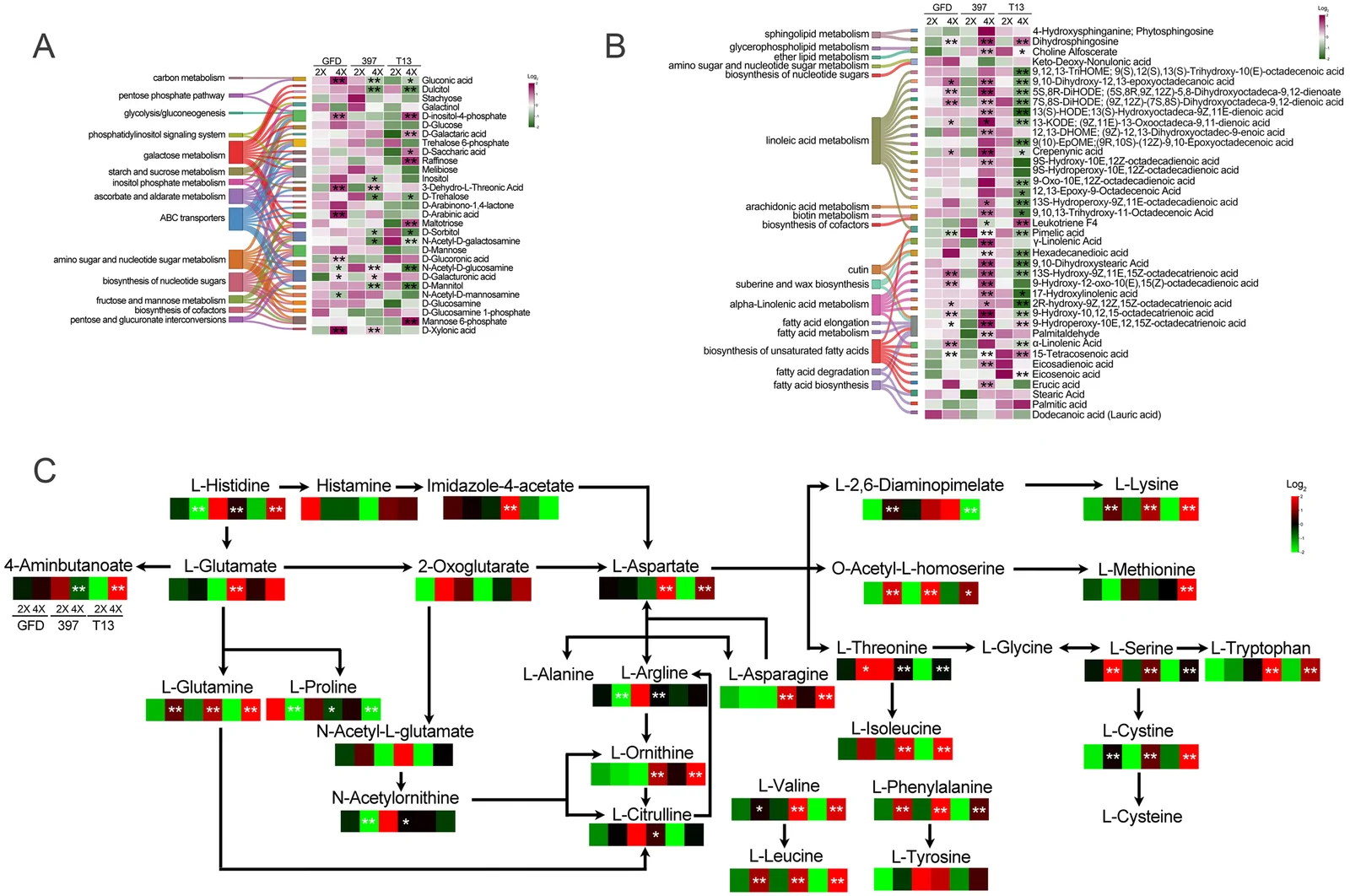
Metabolic alterations in starch, lipid, and protein components. **(A)** Plots display combined Sankey diagrams and heatmaps: Sankey flows represent carbohydrate and saccharide metabolisms associated with enriched KEGG pathways, while heatmaps show expression levels. **(B)** Plots display combined Sankey diagrams and heatmaps: Sankey flows represent lipid metabolisms associated with enriched KEGG pathways, while heatmaps show expression levels. **(C)** Biosynthesis and metabolism of amino acids and derivatives. Asterisks denote significance (*p < 0.05, **p < 0.01).

Among the lipid metabolites, 246 were detected, and 39 were annotated by 16 functional pathways (**Figure 4B**). Visualization of lipid metabolites showed that they mapped to 16 KEGG pathways (**Figure 4B**). Visualization of lipid metabolites showed that they were mainly associated with pathways such as “Fatty acid biosynthesis”, “alpha-Linolenic acid metabolism”, and “Linoleic acid metabolism”. Moreover, most lipid metabolites mapped to these pathways showed significant differences between diploid and autotetraploid brown rice.

Among the annotated saccharide metabolites, the largest number was assigned to “Galactose metabolism”, and the majority of metabolites within this pathway were significantly increased in at least one diploid-autotetraploid comparison. Among the annotated lipid metabolites, the largest number was “Linolenic acid metabolism” pathway. The relative abundances of metabolites in this pathway increased in GFD-4X and 397-4X but decreased significantly in T13-4X. In addition, saccharide metabolites were assigned to pathways including “Biosynthesis of nucleotide sugars”, “Ascorbate and aldarate metabolism”, and “Starch and sucrose metabolism”. Notably, gluconic acid and N-acetyl-D-glucosamine were significantly increased across all three diploid–autotetraploid comparisons.

Together, these results indicate that chromosome doubling altered the relative abundances of carbohydrate- and lipid-related metabolites in brown rice. Saccharide metabolites generally showed higher relative abundances in the autotetraploids, whereas the responses of lipid metabolites were more lines dependent. KEGG pathways related to amino acid biosynthesis, metabolism, and degradation were integrated to construct an amino acid metabolic network based on the relative abundances of the corresponding metabolites (**Figure 4C**). The results revealed significant differences between diploid and autotetraploid brown rice in the relative abundances of essential amino acids, including lysine, phenylalanine, valine, and leucine, of non-essential amino acids, and of several pathway intermediates. In addition, metabolites associated with pathways involving L-glutamine and O-acetyl-L-homoserine showed significant variation. Overall, the metabolomic data were broadly consistent with the targeted biochemical measurements, both indicating higher levels of amino acid-related compounds in autotetraploid than in diploid brown rice. Because the two datasets were generated on different platforms and report relative abundances and absolute concentrations, respectively, they should be regarded as mutually supportive rather than as direct cross-validation.

### 3.5 Integrated metabolic network construction and metabolite level changes in autotetraploid lines

Based on DAMs and pathway enrichment analysis, we constructed an integrated schematic metabolic network (**Figure 5**) centered on tricarboxylic acid (TCA) cycle intermediates (citrate, fumarate, and succinate). This network summarizes the known involvement of these intermediates in carbon and energy metabolism, nitrogen assimilation, and amino acid biosynthesis, and depicts the links between starch-derived sugars and amino acid biosynthesis.

**Figure 5:**
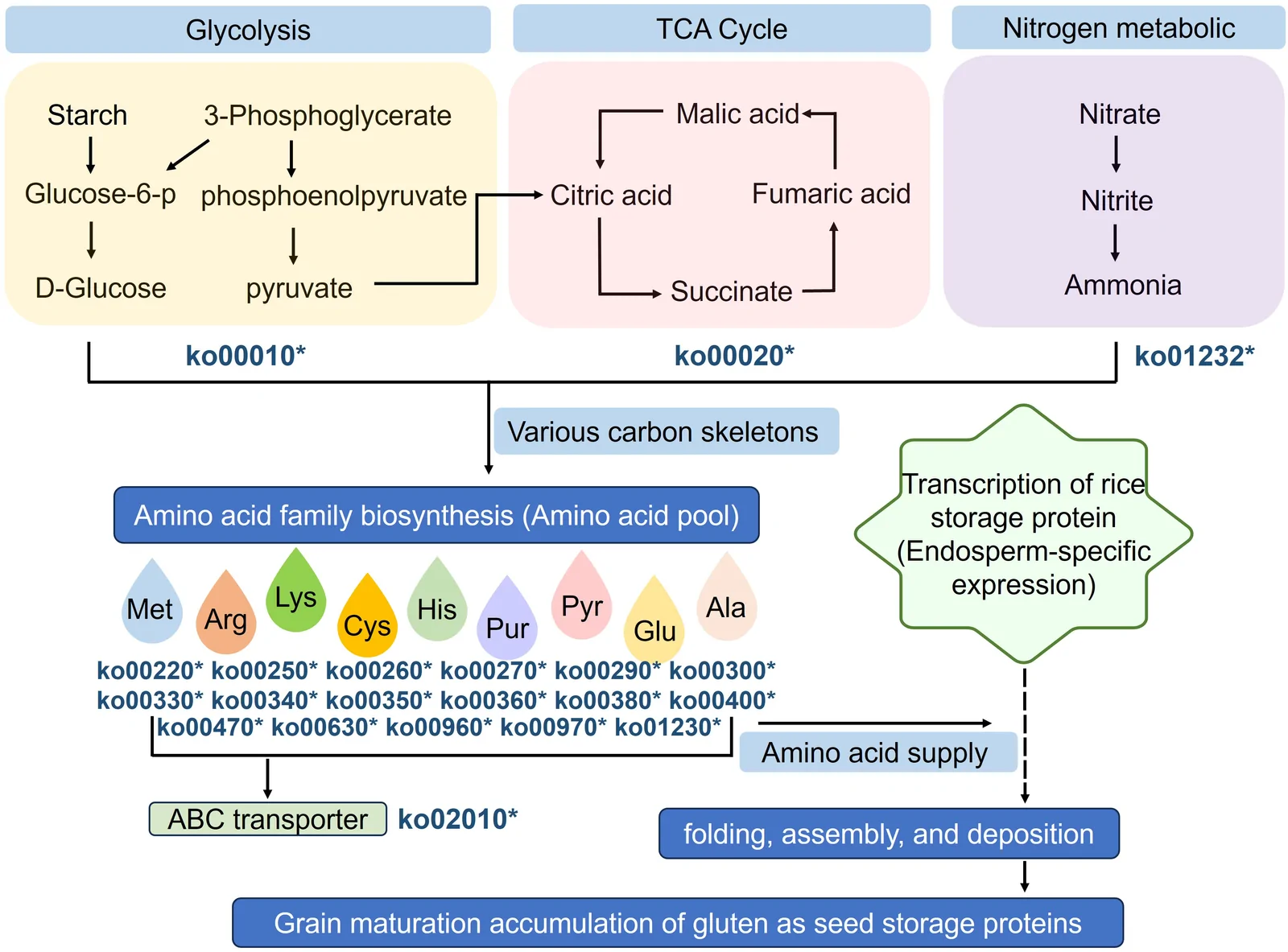
A proposed metabolic network underlying improved nutritional quality in autotetraploid brown rice revealed by differential metabolites. The diagram integrates key DAMs and associated pathways. Solid boxes highlight central organic acids that serve as hubs linking carbon metabolism and amino acid synthesis. Arrows indicate proposed metabolic flux directions or connections. KEGG pathways are labeled with “ko + digit”, asterisks (*) denote significantly enriched pathways compared autotetraploid and diploid rice.

Within the network, starch-derived carbohydrates connect to glycolytic intermediates (e.g., glucose-6-phosphate and pyruvate) and subsequently converge on the TCA cycle. A parallel nitrogen-metabolic module is depicted from nitrate to nitrite and then to ammonium, which is further linked to amino acid synthesis pathways. It should be noted that nitrate, nitrite, and ammonium were not measured in the present metabolomic dataset, this module is therefore presented on the basis of established pathway knowledge rather than on our own measurements. The amino acid biosynthesis module includes multiple branches (e.g., those leading to methionine, lysine, cysteine, and histidine, together with glutamine- and glutamate-related metabolites), and the corresponding metabolite nodes were among the DAMs that showed higher relative abundances in autotetraploid brown rice. The network further places amino acid availability in the context of amino acid transport (ABC transporters), amino-acid-dependent protein processing (folding, assembly, and deposition), and the accumulation of glutelin, the major seed storage protein of rice, during grain maturation. Because transport and protein-processing steps were not assayed in this study, these components of the network are shown as a working model to be tested.

KEGG enrichment analysis showed that DAMs with higher relative abundances in autotetraploid brown rice were significantly enriched in interconnected pathways, including carbon metabolism, nitrogen assimilation, and amino acid biosynthesis, relative to the parental diploids. Enrichment analysis identifies over-representation of DAMs in a pathway and does not by itself demonstrate increased pathway activity or flux. Within these pathways, most-but not all-of the DAMs showed higher relative abundances in the autotetraploids.

## 4 Discussion

Rice nutritional value is largely governed by the composition and abundance of its grain metabolites, yet systematic studies evaluating both nutritional profiles and metabolomic dynamics between diploid and autotetraploid rice remain limited. In this study, targeted chemical analysis, including HPLC and the Kjeldahl method, were integrated with UPLC-MS/MS-based metabolomics to investigate differences in nutrient accumulation and metabolite profiles across three diploid-autotetraploid brown rice pairs. The autotetraploid lines exhibited significantly elevated components of crude fat, crude protein, and most amino acids, along with pronounced shifts in carbohydrate- and lipid-related metabolites. However, ploidy-associated changes were selective rather than uniform: while amino acids and their derivatives were predominantly upregulated, flavonoids exhibited an overall decline, and specific micronutrients such as vitamin D decreased. These results demonstrate that autotetraploidization induces a systematic reprogramming of grain metabolic networks rather than a global amplification of all nutrients.

### 4.1 Chromosome doubling alters plant nutrition-related metabolic profiles

Polyploidization is a recognized evolutionary force and a proven strategy for crop trait improvement (Barker et al., 2024; Cheng et al., 2018). Beyond simply expanding genome dosage, polyploidization triggers extensive physiological remodeling. In vegetative organs of polyploid plants, enhanced photosynthetic pigment content, altered stomatal conductance, and improved chloroplast electron transport have been frequently reported (Šmarda et al., 2023; Zhang et al., 2024; Wu et al., 2025). These physiological adjustments provide a theoretical basis for elevated assimilate generation, however, whether source activity and sink strength are coordinately enhanced in reproductive grains depends heavily on regulatory cascades during grain filling (Zeng et al., 2026).

In secondary metabolism, polyploidization typically promotes the accumulation of flavonoids, phenolics, terpenoids and alkaloids in fruit and vegetative tissues, as observed in *Ziziphus jujuba* (Han et al., 2025) and *Stevia rebaudiana* (Liu et al., 2024). Strikingly, our metabolomic profiling in brown rice revealed an opposite trend: flavonoids showed a widespread decrease in autotetraploids. Across the 13 annotated metabolite categories, differential responses were observed between primary and secondary metabolites, as well as among distinct genotypes. These findings suggest that while certain metabolic responses to whole-genome doubling are consistent across the three varieties tested (e.g., increased amino acids, decreased flavonoids), the magnitude and direction of changes in other metabolite classes are variety-dependent and may be further constrained by tissue type and environmental conditions. Whether these variety-dependent responses can be generalized across additional rice genetic backgrounds and other taxa warrants further investigation.

In this study, interpretations of polyploidization effects should be made with due caution regarding several potential limitations. First, the tetraploid materials used here were generated through different routes: GFD-4X and T13-4X originated from spontaneous tetraploid mutations, whereas 397-4X was produced by colchicine treatment. Although all lines were subsequently subjected to seven consecutive generations of self-pollination at the Teaching and Research Base of Jilin Agricultural University, and phenotypic selection was applied to ensure stability and uniformity of major agronomic traits-procedures that may reduce confounding effects from somaclonal variation and mitigate the influence of the original doubling method on nutritional composition: material-origin differences cannot be completely ruled out.

Critically, the core comparisons in this work were always conducted within each genetic background, specifically by comparing each autotetraploid line with its corresponding isogenic diploid counterpart. It is precisely because the three varieties originated from different induction routes that the consistent nutritional changes observed across these genetic backgrounds provide stronger evidence for an association with increased ploidy level, rather than effects driven by the induction method itself. If differences were primarily attributable to the induction protocol, we would expect the colchicine-induced 397-4X to display a markedly different response pattern from the spontaneously derived GFD-4X/T13-4X. Instead, within our within-lines comparison framework, all three autotetraploids show consistent increases in key nutritional traits, including crude protein, most essential amino acids, and crude fat. This cross-variety concordance indicates that the observed changes are more likely to result from the increase in ploidy level itself, rather than from differences in the specific method of tetraploidization.

Second, the field experimental design also presents certain constraints. A randomized complete block design was adopted with three replicated plots per line and standardized field management (including fertilization, irrigation, and pest control). However, only one tetraploid line was included for each genetic background, which limits the ability to rigorously disentangle polyploid-driven effects from line-specific genetic drift. Moreover, molecular marker- or genome-level assessment of background identity was not performed in this study, restricting our capacity to precisely evaluate the degree of genetic background alignment between each tetraploid line and its diploid progenitor.

Taken together, the comparative framework established within each genetic background provides initial evidence for the effects of rice polyploidization on nutritional traits. In particular, for traits showing consistent trends across three independent genetic backgrounds, the association with ploidy level is relatively stronger. Although tetraploid origins are heterogeneous and the study lacks replicated tetraploid lines per background, the concordant response patterns observed across multiple independent varieties lend support to a ploidy-dependent effect, thereby lending relative credibility to cross-variety generalizations. Future studies should generate multiple independent tetraploid replicates using a uniform doubling protocol and incorporate molecular marker- or whole-genome sequencing-based characterization to enable more rigorous genetic background matching, thereby providing a more comprehensive understanding of the general effects of polyploidization on rice nutritional quality and its potential underlying molecular mechanisms.

### 4.2 Compositional differences and their nutritional implications

Starch constitutes 80%-90% of rice dry weight, with its functional and eating quality determined by amylose and amylopectin architectures (Zhang et al., 2025; Vandeputte and Delcour, 2004). In this study, total amylose concentrations did not differ significantly between ploidy levels. Interestingly, despite the absence of significant changes in total amylose, RS content increased substantially (by more than 26%) in two autotetraploid lines. Because RS formation is not governed solely by gross amylose concentration, this divergence strongly points to structural alterations in starch granules. Autotetraploidization may alter the ratio of intermediate-to-long debranched amylopectin chains (degree of polymerization, DP), promote B-type crystallite formation, or enhance the physical complexation between starch and the elevated lipid/protein pools, thereby shielding starch from enzymatic hydrolysis (Man, 2015; Li et al., 2019; Ying et al., 2022). Although elevated RS represents a desirable low-glycemic dietary trait, its functional efficacy requires validation under standard processing and human digestion simulations.

Protein content (7%-10% of endosperm dry weight, Martin and Fitzgerald, 2002) and most amino acids were significantly higher in autotetraploid grains. Essential amino acids increased by 20%-38%, with prominent increases in lysine and threonine (Zhang and Song, 1992). Because lysine serves as the primary limiting amino acid in cereal endosperm, its marked enhancement substantially improves the potential amino acid balance of brown rice (Shao, 2020). However, metabolomic detection of free or soluble amino acid pools must not be conflated with the overall amino acid profile of storage proteins. True dietary protein quality requires rigorous verification through *in vitro* digestibility and bioavailability assays.

Lipid- and vitamin-related profiles exhibited both favorable changes and nutritional trade-offs. Crude fat content was generally higher in autotetraploids, which could influence starch gelatinization and sensory attributes through lipid-starch complexation (Photinam and Moongngarm, 2023; Yang et al., 2017; Zhang et al., 2023). However, regarding micronutrients, while certain B- and E-related compounds showed positive trends, vitamins D exhibited a notable decrease in autotetraploids. Because the autotetraploid plants exhibited a higher thousand-grain weight, we observed a moderate decrease in the dry-weight-normalized concentrations (mg/g or µg/g) of amylose and vitamin D. This pattern may, at least in part, reflect a “dilution effect”: when grain mass increases, the dry-weight-normalized concentration of a given component will decline if its absolute accumulation per grain does not increase proportionally (or increases less than the degree of grain enlargement). It is important to emphasize that this explanation does not rule out metabolomic reprogramming caused by polyploidization, which could weaken the biosynthetic, conversion, or storage capacity of these compounds and/or change their molecular forms/structures.

In contrast, most amino acids and higher-level nutritional traits such as crude protein and crude fat increased significantly after polyploidization, suggesting that the absolute accumulation of these components likely rose to a greater extent, thereby offsetting the potential dilution associated with increased grain mass. It should also be noted that these changes do not follow a simple, dose-proportional effect, instead, they are strongly influenced by genetic background and show pronounced varieties dependence. Because this study examined only three diploid-autotetraploid genotype pairs, these conclusions apply to the materials tested and cannot be directly generalized to autotetraploid rice as a whole.

### 4.3 KEGG pathway enrichment and integrated metabolic networks

KEGG pathway enrichment revealed distinct metabolic modules responsive to autotetraploidization. Among saccharide metabolites, the highest enrichment occurred in “Galactose metabolism”, alongside significant overrepresentation in “Amino sugar and nucleotide sugar metabolism” and “Starch and sucrose metabolism”. Upregulation of key intermediates, notably gluconic acid and N-acetyl-D-glucosamine across comparisons, indicates heightened turnover in nucleotide sugar biosynthesis, which channels carbon flux toward cell wall polysaccharides and glycoprotein precursors rather than expanding the storage starch pool.

In lipid metabolism, annotated pathways such as “alpha-Linolenic acid metabolism” and “Fatty acid biosynthesis” were significantly enriched. Importantly, metabolite responses in linolenic acid metabolism diverged sharply by genotype-increasing in GFD-4X and 397-4X but declining in T13-4X. This divergence demonstrates that ploidy-driven lipid metabolic shifts are modulated by background-specific regulatory networks.

In nitrogen and amino acid metabolism, KEGG analysis demonstrated coordinated enrichment across lysine biosynthesis, cysteine/methionine metabolism, and the alanine, aspartate, and glutamate pathway. The integrated metabolic network placed tricarboxylic acid (TCA) cycle intermediates (citrate, fumarate, succinate) at central hubs, directly linking central carbon glycolysis to amino acid biosynthesis. Soluble sugars derived from upstream carbohydrate turnover provide the carbon skeletons (e.g., 2-oxoglutarate and oxaloacetate) required for transamination reactions (Foyer et al., 2011; Nunes-Nesi et al., 2013). Nevertheless, because metabolomic data quantify static metabolite pool sizes rather than kinetic reaction rates, KEGG overrepresentation indicates pathway reorganization rather than proven flux acceleration.

### 4.4 Mechanistic discussion of metabolic reprogramming

The contrasting trends between amino acids and flavonoids provide suggestive evidence for carbon metabolic flux redistribution induced by autotetraploidization. The underlying mechanism may originate from “flux trade-off” at shared precursor nodes (Massad et al., 2012). Specifically, the phenylalanine node sits at the junction between primary and secondary metabolism: on one hand, it enters the phenylpropanoid/flavonoid synthesis pathway via phenylalanine ammonia-lyase (PAL), on the other hand, it serves as an essential substrate for the translational synthesis of storage proteins (Fernie and Tohge, 2017). During grain filling, whole-genome doubling may preferentially drive carbon and nitrogen flux toward protein deposition at the sink, resulting in substantial consumption of phenylalanine for amino acid pool expansion and glutelin assembly, thereby competitively reducing substrate availability for the flavonoid branch and ultimately leading to the relative decline in flavonoid abundance.

At the cellular and developmental regulation level, polyploid plants commonly exhibit the “gigas effect,” characterized by increased cell volume, adjusted internal membrane structures, and expanded storage space (Šmarda et al., 2023; Wu et al., 2025; Doyle and Coate, 2019). Such changes in subcellular microstructure may expand the physical storage capacity of the endosperm, providing physical support for the deposition of protein bodies and resistant starch structures. Meanwhile, gene dosage effects and transcriptional reprogramming brought about by whole-genome doubling may synergistically activate key transport proteins (such as ABC transporters) and protein folding pathways, collectively facilitating the establishment of high nutrient storage capacity in grains.

The limitations of this study include the analysis of only three pairs of varieties, so conclusions should not be directly extrapolated to all rice germplasm, furthermore, static endpoint measurements cannot directly reveal the dynamics of grain filling. Future studies combining dynamic multi-timepoint sampling during grain filling, transcriptomics, enzyme kinetics analysis, and isotopic tracer techniques will be necessary to ultimately verify the molecular mechanisms underlying nutrient enrichment and metabolic flux allocation in autotetraploid rice.

## 5 Conclusion

This study systematically compared the nutritional composition and metabolomic profiles of three diploid-autotetraploid rice pairs, revealing that whole-genome doubling profoundly reshapes grain metabolism through a selective reprogramming of nutritional networks, rather than a uniform amplification. Autotetraploid brown rice consistently exhibited elevated levels of crude protein, most essential amino acids, and crude fat. Metabolomic analysis further identified extensive shifts in saccharide, lipid, and amino acid-related metabolites, with KEGG enrichment highlighting increased amino acid biosynthesis pathways. However, these gains were accompanied by specific metabolic trade-offs. Flavonoid metabolites showed a declining trend, and vitamin D content decreased, indicating that polyploidization induces a rebalancing of metabolic resources. While autotetraploid rice shows promise for targeted nutritional improvement, particularly in protein and amino acid content, its utility is constrained by lines dependent responses. Future research, including dynamic multi-timepoint analyses, transcriptomics, and *in vitro*/*in vivo* bioavailability assessments, is essential to fully elucidate its underlying mechanisms and validate true nutritional enhancement for breeding next-generation rice varieties.

## Data Availability

The original contributions presented in the study are included in the article/**Supplementary Material**. Further inquiries can be directed to the corresponding authors.

## Supplementary material

Supplementary Figure 1 Karyotype analysis of the tetraploid rice lines based on metaphase chromosomes in root-tip cells. **(A)** GFD-4X. **(B)**, 397-4X. **(C)**, T13-4X.

Supplementary Figure 2 The nutrient contents of diploid and autotetraploid brown rice was conducted.

Supplementary Figure 3 Quality assessment of metabolomic data. **(A)**, Overlaid total ion chromatograms of pooled QC samples in positive ion modes, respectively. **(B)**, Overlaid total ion chromatograms of pooled QC samples in negative ion modes, respectively. **(C)**, Cumulative distribution of metabolite CV values. **(D)**, PCA score plot of metabolomic profiles, QC represents the quality control group.

Supplementary Figure 4 Correlation analysis in metabolite abundance among the three biological replicates within each brown rice line.

Supplementary Figure 5 The OPLS-DA model of three groups of diploid and autotetraploid brown rice.

Supplementary Figure 6 K-means clustering analysis on DAMs.

Supplementary Figure 7 KEGG enrichment analysis of the identified down-regulated DAMs.

Supplementary Table 1 The thousand seed weight of three groups of diploid and autotetraploid rice.

Supplementary Table 2 The nutritional contents of three groups of diploid and autotetraploid brown rice (results of multiple comparisons are included).

Supplementary Table 3 Classification information of metabolites in three groups of diploid and autotetraploid brown rice metabolomics.

Supplementary Table 4 The information of three groups of diploid and autotetraploid brown rice metabolomics.

Supplementary Table 5 Common differential metabolites in diploid and autotetraploid brown rice.

Supplementary Table 6 KEGG enrichment classifications for different changes in metabolites in three groups of diploid and autotetraploid

## References

[B1] Barker M. S. Jiao Y. Glennon K. L. (2024). Doubling down on polyploid discoveries: global advances in genomics and ecological impacts of polyploidy. Am. J. Bot. 111, e16395. doi: 10.1002/ajb2.16395 39164922

[B2] Cheng F. Wu J. Cai X. Liang J. L. Freeling M. Wang X. W. (2018). Gene retention, fractionation and subgenome differences in polyploid plants. Nat. Plants 4, 258–268. doi: 10.1038/s41477-018-0136-7 29725103

[B3] Comai L. (2005). The advantages and disadvantages of being polyploid. Nat. Rev. Genet. 6, 836–846. doi: 10.1038/nrg1711 16304599

[B4] Dhawan O. P. Lavania U. C. (1996). Enhancing the productivity of secondary metabolites via induced polyploidy: a review. Euphytica 87, 81–89. doi: 10.1007/bf0002187930311153

[B5] Doyle J. J. Coate J. E. (2019). Polyploidy, the nucleotype, and novelty: the impact of genome doubling on the biology of the cell. Int. J. Plant Sci. 180, 1–52. doi: 10.1086/700636 34335698 PMC8318037

[B6] Fernie A. Tohge T. (2017). The genetics of plant metabolism. Annu. Rev. Genet. 51, 287–310. doi: 10.1146/annurev-genet-120116-024640 28876980

[B7] Foyer C. H. Noctor G. Hodges M. (2011). Respiration and nitrogen assimilation: targeting mitochondria-associated metabolism as a means to enhance nitrogen use efficiency. J. Exp. Bot. 62, 1467–1482. doi: 10.1093/jxb/erq453 21282329

[B8] Goufo P. Trindade H. (2017). Factors influencing antioxidant compounds in rice. Crit. Rev. Food Sci. Nutr. 57, 893–922. doi: 10.1080/10408398.2014.922046 25897468

[B9] Gurung P. Zubair M. Jialal I. (2025). “ Plasma glucose,” in Statpearls ( StatPearls Publishing, Treasure Island (FL)). 31082125

[B10] Han Y. Sui J. Yao T. Chen S. Yang M. He M. . (2025). Phenotypic and metabolic variations induced by autopolyploidization in Chinese jujube cultivars. Plants 14, 3588. doi: 10.3390/plants14233588 41375303 PMC12694531

[B11] Kasote D. Sreenivasulu N. Acuin C. Regina A. (2022). Enhancing health benefits of milled rice: current status and future perspectives. Crit. Rev. Food Sci. Nutr. 62, 8099–8119. doi: 10.1080/10408398.2021.1925629 34036858

[B12] Khalid H. Amin F. R. Chen C. (2021). An integrated laboratory experiment for the determination of main components in different food samples. Biochem. Mol. Biol. Educ. 50, 133–141. doi: 10.1002/bmb.21592 34897926

[B13] Leng Z. T. Liu K. Y. Wang C. X. Qi F. Zhang C. Y. Li D. Y. . (2023). A comparative analysis of major cell wall components and associated gene expression in autotetraploid and its donor diploid rice (*Oryza sativa* L.) under blast and salt stress conditions. Plants 12, 3976. doi: 10.3390/plants12233976 38068612 PMC10708163

[B14] Li H. Gidley M. J. Dhital S. (2019). High-amylose starches to bridge the “fiber gap”: development, structure, and nutritional functionality. Compr. Rev. Food Sci. Food Saf. 18, 362–379. doi: 10.1111/1541-4337.12416 33336945

[B15] Liu J. Wang J. Chen M. Meng W. Ding A. Chen M. . (2024). Multi-omics analyses uncover the mechanism underlying polyploidization-enhanced steviol glycosides biosynthesis in *Stevia rebaudiana*. Plants 13, 2542. doi: 10.3390/plants13182542 39339518 PMC11434884

[B16] Liu Z. D. Wang J. Y. Li L. Wu P. (2021). Mechanistic insights into the role of starch multi-level structures in functional properties of high-amylose rice cultivars. Food Hydrocolloids 113, 106441. doi: 10.1016/j.foodhyd.2020.10644142574925

[B17] Liu L. Waters D. L. E. Rose T. J. Bao J. S. King G. J. (2013). Phospholipids in rice: significance in grain quality and health benefits: a review. Food Chem. 139, 1133–1145. doi: 10.1016/j.foodchem.2012.12.046 23561219

[B18] Man J. M. (2015). Structural and Characteristic Studies of Endosperm Starch Granules in High-Amylose rice dissertation (Yangzhou: Yangzhou University).

[B19] Martin M. Fitzgerald M. A. (2002). Proteins in rice grains influence cooking properties. J. Cereal Sci. 36, 285–294. doi: 10.1006/jcrs.2001.046521632986

[B20] Massad T. J. Dyer L. A. Vega C. G. (2012). Costs of defense and a test of the carbon-nutrient balance and growth-differentiation balance hypotheses for two co-occurring classes of plant defense. PloS One 7, e47554. doi: 10.1371/journal.pone.0047554 23115654 PMC3480385

[B21] Nunes-Nesi A. Araújo W. L. Obata T. Fernie A. R. (2013). Regulation of the mitochondrial tricarboxylic acid cycle. Curr. Opin. Plant Biol. 16, 335–343. doi: 10.1016/j.pbi.2013.01.004 23462640

[B22] Photinam R. Moongngarm A. (2023). Effect of adding vegetable oils to starches from different botanical origins on physicochemical and digestive properties and amylose-lipid complex formation. J. Food Sci. Technol. 60, 393–403. doi: 10.1007/s13197-022-05626-7 36618064 PMC9813310

[B23] Saleh A. S. M. Wang P. Wang N. Yang L. Xiao Z. G. (2019). Brown rice versus white rice: nutritional quality, potential health benefits, development of food products, and preservation technologies. Compr. Rev. Food Sci. Food Saf. 18, 1070–1096. doi: 10.1111/1541-4337.12449 33336992

[B24] Sattler M. C. Carvalho C. R. Clarindo W. R. (2016). The polyploidy and its key role in plant breeding. Planta 243, 281–296. doi: 10.1007/s00425-015-2450-x 26715561

[B25] Shao Y. F. (2020). Nutritional and functional characteristics of rice grain. China Rice 26, 1–11. doi: 10.3969/j.issn.1006-8082.2020.06.001

[B26] Shen L. S. Li J. Y. Li Y. H. (2022). Resistant starch formation in rice: genetic regulation and beyond. Plant Commun. 3, 100329. doi: 10.1016/j.xplc.2022.100329 35576157 PMC9251435

[B27] Shewry P. R. (2007). Improving the protein content and composition of cereal grain. J. Cereal Sci. 46, 239–250. doi: 10.1016/j.jcs.2007.06.00642574925

[B28] Šmarda P. Klem K. Knápek O. Veselá B. Veselá K. Holub P. . (2023). Growth, physiology, and stomatal parameters of plant polyploids grown under ice age, present-day, and future CO_2_ concentrations. New Phytol. 239, 399–414. doi: 10.1111/nph.18955 37167007

[B29] Tappiban P. Ying Y. Xu F. F. Bao J. S. (2021). Proteomics and post-translational modifications of starch biosynthesis-related proteins in developing seeds of rice. Int. J. Mol. Sci. 22, 5901. doi: 10.3390/ijms22115901 34072759 PMC8199009

[B30] Vandeputte G. E. Delcour J. A. (2004). From sucrose to starch granule to starch physical behaviour: a focus on rice starch. Carbohydr. Polym. 58, 245–266. doi: 10.1016/j.carbpol.2004.06.00342574925

[B31] Van Drunen W. E. Johnson M. T. J. (2022). Polyploidy in urban environments. Trends Ecol. Evol. 37, 507–516. doi: 10.1016/j.tree.2022.02.005 35246321

[B32] Wang W. Tu Q. Chen R. R. Lv P. C. Xu Y. Q. Xie Q. . (2022). Polyploidization increases the lipid content and improves the nutritional quality of rice. Plants 11, 132. doi: 10.3390/plants11010132 35009135 PMC8747249

[B33] Weh K. M. Howard C. L. Zhang Y. Tripp B. A. Clarke J. L. Howell A. B. . (2024). Prebiotic proanthocyanidins inhibit bile reflux-induced esophageal adenocarcinoma through reshaping the gut microbiome and esophageal metabolome. JCI Insight 9, e168112. doi: 10.1172/jci.insight.168112 38329812 PMC11063939

[B34] Wu Y. N. Zheng Y. X. Yang W. C. Liu N. W. Pang X. M. Li Y. Y. (2025). Unveiling enhanced photosynthesis in tetraploid sour jujube through transcriptome analysis. N. Z. J. Crop Hortic. Sci. 53, 1447–1462. doi: 10.1080/01140671.2024.235887737339054

[B35] Yang Z. Han X. Zhang L. J. Wu H. Y. Liu W. T. (2017). Progress on anti-retrogradation technique of rice products. Food. Ind. 38, 258–262.

[B36] Ying Y. Zhang Z. Tappiban P. Xu F. Deng G. Dai G. . (2022). Starch fine structure and functional properties during seed development in BEIIb active and deficient rice. Carbohydr. Polym. 292, 119640. doi: 10.1016/j.carbpol.2022.119640 35725200

[B37] Yoshida H. Tomiyama Y. Mizushina Y. (2010). Lipid components, fatty acids and triacylglycerol molecular species of black and red rices. Food Chem. 123, 210–215. doi: 10.1016/j.foodchem.2010.04.01042574925

[B38] Zeng Y. Yang J. Gao Y. Tan M. Lin Z. Xiao Z. . (2026). T6P-mediated source-sink coordination regulates grain filling of rice inferior spikelets. J. Integr. Agric. 25. doi: 10.1016/j.jia.2026.06.01842574925

[B39] Zhang R. Rao S. Wang Y. Qin Y. Qin K. Chen J. (2024). Chromosome doubling enhances biomass and carotenoid content in *Lycium chinense*. Plants 13, 439. doi: 10.3390/plants13030439 38337972 PMC10857560

[B40] Zhang Y. H. Song W. C. (1992). Rice tetraploidy and its effect on agronomic traits and nutritional constituents. Acta Agron. Sin. 18, 137–144.

[B41] Zhang H. B. Tang S. Q. Wei F. N. Zong W. B. Hou J. B. Ran X. . (2025). The effects of photoperiodic transcription factor OsPRR37 on grain filling and starch synthesis during rice caryopsis development. Plants 14, 3690. doi: 10.3390/plants14233690 41375399 PMC12694350

[B42] Zhang C. N. Xue W. Li T. Wang L. (2023). Understanding the relationship between the molecular structure and physicochemical properties of soft rice starch. Foods 12, 3611. doi: 10.3390/foods12193611 37835265 PMC10572268

[B43] Zhou K. Luo Z. L. Huang W. D. Liu Z. M. Miao X. X. Tao S. H. . (2024). Biological roles of lipids in rice. Int. J. Mol. Sci. 25, 9046. doi: 10.3390/ijms25169046 39201734 PMC11354756

[B44] Zhu J. H. Tao K. Y. Prakash S. Zhang C. Q. Gilbert R. G. Liu Q. Q. (2023). Using starch structure to choose rices with an optimal combination of palatability and digestibility. Food Hydrocolloids 141, 108763. doi: 10.1016/j.foodhyd.2023.10876342574925

